# A Two-Dimensional (2-D) Sensor Network Architecture with Artificial Intelligence Models for the Detection of Magnetic Anomalies

**DOI:** 10.3390/s26030764

**Published:** 2026-01-23

**Authors:** Paolo Gastaldo, Rodolfo Zunino, Alessandro Bellesi, Alessandro Carbone, Marco Gemma, Edoardo Ragusa

**Affiliations:** 1 Department of Naval, Electrical, Electronic and Telecommunications Engineering (DITEN), University of Genoa, 16145 Genoa, Italy; paolo.gastaldo@unige.it (P.G.); rodolfo.zunino@unige.it (R.Z.); 2 Skytech srl, 19136 La Spezia, Italy; bellesi.a@skytechnologies.it (A.B.); carbone.a@skytechnologies.it (A.C.); gemma.m@skytechnologies.it (M.G.)

**Keywords:** magnetic anomaly detection, deep learning, magnetometric networks

## Abstract

The paper presents the development and preliminary evaluation of a two-dimensional (2-D) network of magnetometers for magnetic anomaly detection. The configuration significantly improves over the existing one-dimensional (1-D) architecture, as it enhances the spatial characterization of magnetic anomalies through the simultaneous acquisition of data over an extended area. This leads to a reliable estimation of the target motion parameters. Each sensor node in the network includes a custom-designed electronic system, integrating a biaxial fluxgate magnetometer that operates in null mode. Deep learning models process the raw measurements collected by the magnetometers and extract structured information that enables both automated detection and preliminary target tracking. In the experimental evaluation, a 5×5 array of nodes was deployed over a 12×12 m^2^ area for terrestrial tests, using moving ferromagnetic cylinders as targets. The results confirmed the feasibility of the 2-D configuration and supported its integration into intelligent, real-time surveillance systems for security and underwater monitoring applications.

## 1. Introduction

The protection of critical infrastructures has represented a significant challenge for a long time. The detection of underwater threats in ports and coastal areas poses major problems, as high background noise, acoustic shadow zones, or the presence of natural or artificial obstacles hinder conventional acoustic surveillance. In this context, the work of Soldani et al. [1] boosted the development of self-informed magnetometric sensor networks for underwater intrusion detection. That research described a one-dimensional (1-D) array of magnetometers deployed along the seabed and aimed at detecting intrusive targets; each sensor measured both the background magnetic field (natural plus artificial) and the superimposed signal generated by an intruder. The sensors should be properly spaced, close enough to capture correlated background noise, yet sufficiently apart to avoid detecting the same target simultaneously. With that setting, the system can extract the presence of a threat even under adverse ambient noise conditions. Earlier studies by Faggioni et al. [2,3] applied magnetometric networks in harbour environments, featuring real-time noise subtraction and threshold-based intrusion detection.

In spite of those promising results, the 1-D linear architecture exhibits some inherent limitations. When the monitored area has a complex geometry and the intruding object can move in multiple directions, a straightforward linear network provides limited coverage, affected by blind spots or critical regions with reduced sensitivity. Moreover, data processing in previous implementations largely depended on noise subtraction and relied on predefined thresholds.

The research presented in this paper overcomes the 1-D configuration and supports a two-dimensional (2-D) magnetometric network architecture. The transition from a linear to a planar sensor layout yields richer spatial information about the detected magnetic anomalies, including the approximate direction of motion and the relative velocity of the magnetic source. In general, distributed sensing approaches based on sensor networks may suffer from system-level drawbacks such as communication reliability issues, node failures, and vulnerability to environmental disturbances. In order to mitigate these aspects, the proposed system is deliberately based on a non-wireless sensor network with centralized synchronization and wired communication. This architectural choice reduces several limitations commonly discussed in the context of wireless sensor networks [4,5], while preserving the benefits of spatially distributed sensing.

In addition to the architectural evolution, the paper introduces artificial intelligence (AI) models into the data processing pipeline [6,7]. Some inherent limitations affect conventional signal-processing algorithms when extracting structured information from complex, time-varying magnetic data [8,9]. AI methods, such as deep learning (DL) models trained on spatio-temporal magnetic patterns, allow higher-level interpretation of the measured signals, including event classification. This integration of data-driven analysis into a spatially extended sensing network opens new vistas on intelligent, adaptive, and autonomous magnetometric detection systems [10].

Unlike the existing approach [1], which was mostly aimed at underwater deployment, the 2-D configuration defines a general-purpose sensing framework. As a result, the associated implementation and experimental validation can proceed under controlled terrestrial conditions. The ultimate goal is to assess the method’s effectiveness in detecting and possibly tracking moving ferromagnetic objects. The experimental session involved a 5×5 array of sensor nodes.

Ferromagnetic objects have been used to emulate the magnetic signatures of moving metallic bodies, whose signals always superimpose to the background magnetic field. The experimental dataset has been collected by manually moving the objects along predefined paths over the sensor network. Experimental results proved that DL-based models could successfully support both anomaly detection (involving an unsupervised learning problem) and the event detection task (a multiclass categorization problem).

The main contributions of this work can be summarized as follows:

- **Design and implementation of a 2-D magnetometric network:** The paper introduces a novel two-dimensional architecture derived from previously developed 1-D sensor chains. The structure supports the spatially distributed acquisition of the magnitude (field intensity) of the magnetic field over an extended area; this enables the extraction of richer spatial information for event localization and tracking. The complete system has been designed and realized, including the dedicated electronic boards for each sensor node and the distributed power and communication infrastructure.
- **Development of intelligent data-driven processing models:** Data-driven artificial intelligence models process the magnetic field data collected by the network. The resulting framework can detect magnetic anomalies and classify events based on the spatio-temporal patterns in the sensor data, thus extending the capabilities of conventional approaches.
- **Experimental validation on a 5 × 5 terrestrial network:** A comprehensive experimental campaign was carried out on a 5×5 array covering a 12×12 m^2^ area. The results demonstrated the feasibility of the 2-D architecture and its potential for future applications in real-time intrusion detection and environmental monitoring.

In the following, Section 2 reviews the main approaches to harbour protection and magnetic-based intrusion detection, and summarizes the methodology and operating principles of the 1-D system [1]; Section 3 describes the 2-D network architecture and the design of the sensing nodes; Section 4 deals with the deep learning architectures used in the data-processing pipeline; Section 5 presents the experimental setup; Section 6 analyzes the outcomes of the measurement sessions; and Section 7 and Section 8 make concluding remarks and outline possible extensions of the 2-D concept to underwater applications.

## 2. Background

### 2.1. Related Works

The protection of harbour infrastructures from underwater intrusions has been the subject of extensive research, leading to the development of various sensor networks and detection systems in complex marine environments [11,12,13,14,15].

Acoustic sensors, mostly including sonar systems, have been widely used for underwater intrusion detection. Active sonar systems emit sound waves and analyze the echoes received to detect objects in the water. These set-ups offer 360-degree coverage and can detect intruders at ranges of up to 800 m. Environmental factors, however, may affect their performances significantly. Reverberation is a crucial example, involving the sum of waves generated in the volume of water at a certain time by numerous irregular scatters from the sea floor [16]. Acoustic systems remain a cornerstone of underwater surveillance, thanks to their maturity and effectiveness in various conditions [17,18,19,20,21].

Magnetic sensor-based architectures have emerged as a promising complement to acoustic systems, especially whenever acoustic signals may prove unreliable or ineffective. Magnetic sensors detect the disturbances in the Earth’s magnetic field brought about by ferromagnetic objects. These systems are less susceptible to environmental noise and can operate effectively in areas with high acoustic noise [22,23]. The MAC system by Faggioni et al. [2] is a notable example of magnetometric sensors used to detect underwater intrusions: the system relies on a network of fluxgate magnetometers to monitor perturbations in the magnetic field. This provides a sensitive instrument to detect moving ferromagnetic objects in the vicinity of harbour structures. More recently, Soldani et al. introduced the “LFurthermore, / MArine magnetometric detector for self informed system” (LAMA system), a self-informed magnetometer network designed to detect underwater threats in port environments [1]. That system processed a pair of inputs, namely, the environmental magnetic noise and the superimposed signal generated by a moving target. The frequencies of a diver’s signal lie within the noise band, making frequency filtering inadequate for noise removal. The LAMA system addresses that issue by applying advanced signal processing techniques to isolate and identify the target signal from the background noise.

The work by Adamczyk et al. [24] provided a significant advance, relying on an underwater magnetic sensor network (UMSN) specifically engineered for intrusion detection in challenging aquatic environments, such as wet ground and shallow water beds. That system relied on a one-dimensional array of wireless nodes, including AMR-based magnetic sensors integrated with MEMS accelerometers. The design required extensive calibration procedures to correct for both hard-iron and soft-iron distortions, and to ensure accurate field measurements even under varying environmental conditions.

### 2.2. Self-Informed Magnetometric Sensor Networks for Underwater Intrusion Detection

The system presented by Soldani et al. [1] involves a distributed magnetometric network specifically aimed to detect underwater intrusions in harbour and coastal environments. The method relied on a one-dimensional (1-D) array of magnetometers that formed an intelligent sensing barrier. Each node continuously measures the local magnetic field vector and communicates its data to a centralized processing unit. That unit integrates the information from all sensors to point out the transient magnetic anomalies associated with moving ferromagnetic objects.

The detection principle of the system relies on the spatial and temporal correlation of the magnetic field measurements collected by the 1-D network. In marine environments, the ambient geomagnetic field undergoes a combination of natural effects (e.g., diurnal fluctuations, geomagnetic storms) and anthropogenic sources (e.g., noise from nearby vessels or electrical systems). These contributions are largely coherent over spatial scales comparable to sensor spacing. By contrast, the magnetic signature of a localized intruder—such as a diver carrying metallic equipment or an underwater vehicle—produces a short-lived transient, standing out as a spatially confined disturbance.

**Figure 1:**
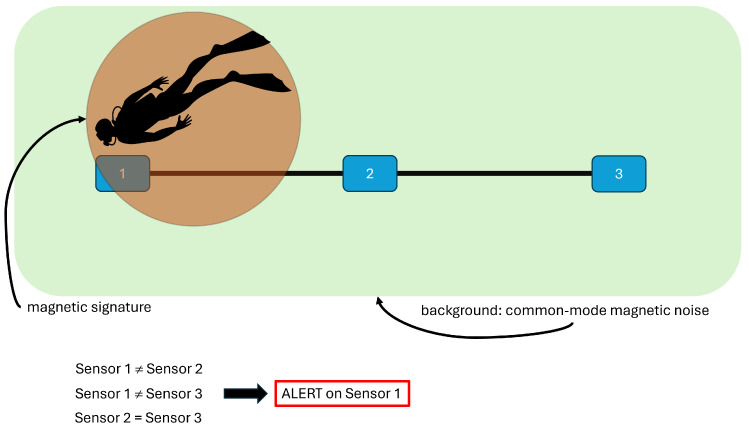
Schematic representation of the 1-D magnetometric sensor network described in [1].

The system pinpoints discrepancies by comparing the magnetic field measurements gathered from adjacent sensors. A differential or correlation-based algorithm removes the common-mode background and highlights local anomalies. As a result, the network can operate effectively even in the presence of strong and variable magnetic backgrounds. The residual signals represent the deviations from the expected geomagnetic pattern, and are analyzed in real time to determine whether they exceed suitably calibrated detection thresholds. Figure 1 illustrates this concept, and shows the linear deployment of magnetometric nodes, the flow of data toward the central processing unit, and the decision logic used for event identification.

The sensors are typically deployed along a fixed baseline on the seabed. The inter-sensor spacing results from a tradeoff between a pair of contrasting requirements. First of all, the deployment should preserve a sufficient correlation of the background magnetic noise between nodes; at the same time, the displacement between sensors should ensure an adequate spatial resolution to localize magnetic anomalies. Spacing values are set experimentally, and depend locally on the magnetic noise spectrum and the expected size of the target objects.

## 3. Two-Dimensional (2-D) Magnetometric Sensor Network for Anti-Intrusion Systems

### 3.1. Two-Dimensional Architecture

The 1-D network of magnetometers described in [1] showed that combining distributed magnetic sensing with spatial noise rejection could support underwater intrusion detection reliably. A 1-D configuration can effectively detect the magnetic anomalies associated with underwater intrusions, but its linear geometry inherently limits the spatial information about the detected target. A 1-D partial observation of the magnetic field makes it difficult to estimate the direction of motion, the trajectory, or the velocity of the intruder.

The two-dimensional (2-D) magnetometric network architecture overcomes these limitations. Sensing nodes span a surface rather than a line, thus forming a bidimensional grid that yields a multi-directional coverage of the monitored area. The 2-D spatial layout allows mapping the magnetic perturbations that mark a moving object; enriching the information content notably improves the ability to localize and characterize the source of the disturbance.

To estimate the relative direction of motion of a ferromagnetic object and track its displacement over time, the system takes into account the spatial gradients of the measured magnetic field across both axes of the network. The additional dimension also enhances the system’s robustness to local magnetic inhomogeneities or partial sensor failures, as a redundancy in spatial sampling allows reliable field interpolation and cross-validation of measurements among neighboring nodes.

**Figure 2:**
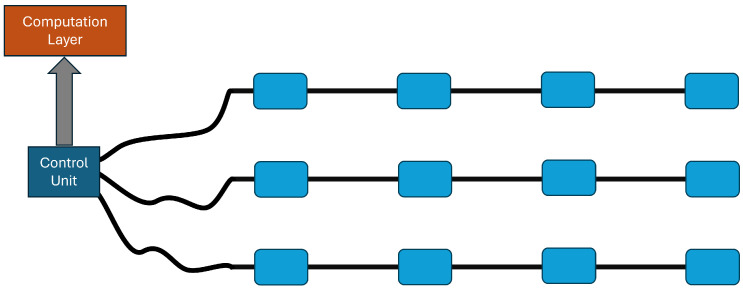
Conceptual schematic of the proposed 2-D magnetometric network.

Figure 2 outlines the 2-D sensor layout and data flow; each node operates as a smart measurement unit, capable of pre-processing local data and transmitting relevant features to a central computation facility. The 2-D network architecture conceptually relies on 1-D magnetometric chains. Each 1-D chain includes an array of magnetometric nodes, serially connected via a dedicated cable along one communication/power line. The chain elements connect to a Control Unit that provides power supply and collects the acquired data. In the one-dimensional sensing chain, data are transmitted sequentially along the chain toward the Control Unit through the wired communication link. This configuration greatly simplifies the underwater installation and the reliability in data transmission, thanks to the centralized control and synchronization.

The upgrade to a 2-D network is achieved by deploying multiple 1-D chains in parallel over the covered area, each chain maintaining its independent wiring to the central acquisition unit. By arranging the chains along two orthogonal (or quasi-orthogonal) directions, one obtains a grid-like structure, which enables the measurement of magnetic field variations in two spatial dimensions. The modular nature of the design allows us to scale the network size and resolution easily, by adding or removing chains according to the expected coverage.

The central unit collects the synchronized data streams from all chains, and builds two-dimensional maps of the magnetic field in real time. This approach preserves the operational simplicity of the 1-D configuration while significantly enhancing the spatial awareness and diagnostic capabilities of the network.

### 3.2. The Sensing Node

Each node in the 2-D magnetometric network operates as an autonomous unit; it can measure the local magnetic field and transmit the acquired data to the central processing unit. The development of the sensor node followed the design principles established during the realization of the 1-D magnetometric network [1], paying particular attention to modularity, long-term stability, and data quality.

The magnetic field measurement stems from a biaxial fluxgate sensor. This choice complies with the experimental observations [1], which demonstrate that for the purpose of intrusion detection and magnetic anomaly mapping, one can measure two components of the magnetic field: one perpendicular to the monitored surface (*z* component) and one longitudinal, i.e., along the axis connecting adjacent nodes within a chain (*x* component). As opposed to a full triaxial setup, a two-axis configuration can therefore yield the necessary spatial information while reducing system complexity, power consumption, and calibration requirements.

**Figure 3:**
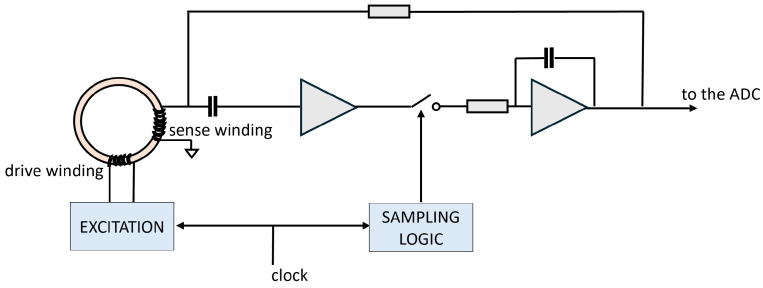
Fluxgate used as a null detector.

The fluxgate sensor operates in *null mode* to obtain a high-quality, low-noise measurement. Figure 3 illustrates the readout circuit. A synchronous detector (or analog multiplier) detects the even harmonics; an analog integrator combines those signals to work out a voltage level that represents the environment magnetic field in the core. This quantity is then integrated and fed back to a compensation coil surrounding the magnetic core. The feedback loop generates a magnetic field that counteracts the external field, effectively nullifying the net magnetic flux inside the core. Under these conditions, the output signal required to maintain the null state is directly proportional to the external magnetic field component being measured. This operating mode offers several advantages, namely improved linearity, faster dynamic response, and reduced core hysteresis, and ultimately increases the precision and stability of the field measurements. The sensor node circuitry includes all the readout electronics to support the fluxgate operation.

On the same board, a microcontroller unit carries out the analog-to-digital conversion of the fluxgate signals and manages communication with the Central Unit. The microcontroller also performs basic tasks such as timing control, synchronization, and sensor diagnostics. A wire-based interface supports the communication with the Central Unit, to ensure robust data transmission and power delivery along the same line. That approach allows the network to operate continuously with precise synchronization across all nodes, enabling a coherent analysis of spatial magnetic field variations.

The resulting sensor architecture is compact, low-power, and mechanically robust, making it suitable for scalable deployment in both terrestrial and underwater configurations. The modularity of the electronic design allows easy adaptation of the interface or communication protocol, if required, to varying experimental setups or environmental conditions.

## 4. Integration of Artificial Intelligence Models

The integration of deep learning models in the data processing chain represents a crucial novel aspect of the overall approach. As a matter of fact, conventional signal processing techniques—such as differential filtering, thresholding, and correlation-based noise rejection—often prove ineffective at either interpreting the underlying cause of the disturbance or extracting higher-level situational information.

AI-based models allow the system to process the spatial and temporal patterns of the magnetic field variations in a more structured manner. Deep learning architectures are trained to identify characteristic signatures of different types of intrusions, distinguishing between various classes of magnetic events. In the 2-D sensor architecture, those models lie in the computational layer, where data converge from the overall network.

According to the setup presented in Section 3, the raw input from the 2-D network is a sequence of R×C matrices, where *R* and *C* are the rows and columns in the 2-D grid, respectively. In the following, $M_{t}\in R^{R\times C}$ will denote the matrix collecting, for each node, the *z* component of the magnetic field sampled at time *t*. As discussed in [1], the *z* component is indeed the most informative source to detect the anomalies induced by a target’s transit. In the present approach, the input for the DL-based models is obtained by building three-dimensional tensors that aggregate the sequence of *T* matrices that starts with $M_{t}$ and ends with $M_{t+T-1}$. As a result, $T_{t}\in R^{R\times C\times T}$ will denote such a tensor.

The paper presents two solutions, which address a pair of different tasks. The first task implies an anomaly detection method, which only aims at identifying significant distortions in the monitored area; distortions mark an intrusion. The second task aims at estimating the direction and the position of the target inside the monitored area. This ultimately leads to a classification task.

In principle, the number of admissible neural network architectures toward that end is huge. An inspection of the input space, however, can help pinpoint the most promising candidates. Indeed, the input data exhibit a strong geometric structure thanks to the specific layout of the sensor network. Moreover, this task does not require deep semantic reasoning across multiple abstraction layers and mainly involves noise filtering and pattern detection. An approach based on convolutional neural networks (CNNs) therefore seems particularly adequate, as they excel at capturing local geometric relationships and suppressing noise.

Temporal relationships are geometrically encoded in the input data: channels represent time frames and are ordered along the third mode of the tensor (*T*). Time series with this structure can be modeled using convolutional, recurrent, or transformer-based networks. However, one considers that the temporal dependencies of interest are short-term; hence CNNs are expected to prove effectively and, at the same time, to alleviate the training complications associated with sophisticated architectures.

### 4.1. Anomaly Detection

The anomaly detection task is approached in an unsupervised fashion and relies on a baseline autoencoder trained on an intrusion-free dataset. The network operation proceeds in two steps: first, it compresses the data and maps them into a latent space, characterized by a much smaller number of dimensions than the original input space.

(1) $$h=f_{enc}\left(x\right)$$

Secondly, in the autoencoder output step, the compressed representation feeds the network and drives the reconstruction of the original data.

(2) $$\tilde{x}=f_{dec}\left(h\right)$$

Autoencoders are usually trained to minimize the reconstruction error, that is, the difference between the original input *x* and the reconstructed pattern $\tilde{x}$. The rationale behind this procedure is to force the network to work out a compressed, latent representation *h* that spans all the training data, thereby learning a concept of “normality” from the data.

The training process involves a training set $T_{tr}=\left\{T_{t};t=1,\ldots ,N_{tr}\right\}$, where $N_{tr}$ denotes the number of tensors collected by processing a sequence of acquisitions in the *no-event* state, i.e., in the absence of any target. Thus, the threshold value that rules the concept of normality stems from the distribution of reconstruction error $E_{r}$, which is defined as follows:

(3) $$E_{r}=\left|T-\tilde{T}\right|$$

The threshold value $t_{nor}$ corresponds to the *p*th percentile of the distribution of $E_{r}$ over the training set $T_{tr}$, where *p* is a parameter to be set in the experimental setup. Then, every new input $T_{t}$ not belonging to the training set will be classified as abnormal (i.e., a significant event) only if $E_{r}>t_{nor}$.

Table 1 outlines the overall neural architecture. The network follows a conventional encoder–latent–decoder structure, specifically tailored to the spatio-temporal nature of the input tensors collected by the 2-D magnetometric network. The table follows the layer order: the first row receives the network inputs and propagates the information up to the last row. The three main blocks refer to the encoder, the fully connected layer for the latent space (as per (1)), and the decoder (2).

The encoder block consists of a sequence of 3D convolutional layers, batch normalization stages, and pooling operations. These layers progressively reduce the spatial dimensions of the input while extracting local spatio-temporal features associated with normal magnetic field variations. The use of small convolutional kernels and a limited number of filters reflects the relatively low complexity of the patterns to be modeled and helps prevent overfitting. The compressed representation produced by the encoder is mapped into a low-dimensional latent space through a fully connected layer. This latent space captures the essential characteristics of the intrusion-free magnetic background and provides a compact representation of normal operating conditions. The decoder mirrors the encoder structure and reconstructs the input tensor from the latent representation using dense, reshaping, upsampling, and convolutional layers. During training, the autoencoder learns to accurately reconstruct normal magnetic patterns, while abnormal events result in increased reconstruction error, which is exploited for anomaly detection.

The table gives, for each layer, the main parameters: $f_{s}$ is the filter size, $k_{s}$ is the kernel size, *h* denotes the dimension of the latent space, and $n_{h}$ gives the units in a dense layer. The architecture aims to limit the amount of parameters and to minimize kernel size. An empirical analysis confirmed that networks with similar topologies but larger number of parameters would not yield significant improvements.

### 4.2. Event Classification

The straightforward anomaly detection approach identifies the presence of an intrusion but does not provide any information about either the nature of the event or the target heading. To address this limitation, a subsequent deep learning model classifies events according to their trajectory across the 2-D network. In this context, an *event* is defined as the transit of a target over the sensing grid along one of the admissible paths considered in the experimental campaign. Let C={1,2,…,E} denote the set of possible classes, where *E* depends on the number of distinct event types to be recognized (e.g., different traversal directions or positions).

The classifier takes as input the same tensors $T_{t}\in R^{R\times C\times T}$ used for anomaly detection. Since these tensors encode both spatial and short-term temporal patterns, the same design principles previously discussed still apply: convolutional architectures remain highly suitable due to their ability to exploit local geometric relationships and suppress noise. Moreover, since anomaly detection and event classification are closely related tasks, the classification network can reuse the encoder structure of the autoencoder.

In more detail, the classifier architecture mirrors the convolutional and upsampling blocks of the autoencoder’s encoder, leveraging their ability to extract hierarchical spatio-temporal features from $T_{t}$. A fully connected layer with $n_{c}$ neurons (where $n_{c}=E$) lies on top of the encoder output. A subsequent softmax activation gives a probability distribution over the event classes. In summary, for an input tensor $T_{t}$, the classifier output is

(4) $$\hat{y}=\mathrm{softmax}\;\left(f_{\mathrm{FC}}\left(f_{\mathrm{enc}}\left(T_{t}\right)\right)\right),$$

where $f_{\mathrm{enc}}$ denotes the convolutional encoder and $f_{\mathrm{FC}}$ the final dense layer. This design choice ensures architectural coherence across the two tasks, reduces the total number of parameters, and simplifies training by exploiting feature extractors already optimized for reconstructing normal magnetic patterns.

Although the classifier architecture shares the same convolutional feature extraction structure as the encoder described above, the event-classification network is trained independently and end-to-end using labeled event data. In particular, no pre-trained weights from the anomaly-detection autoencoder are reused during classifier training. In the training phase, the classifier processes a dataset of labeled event sequences, and each tensor $T_{t}$ marks the trajectory performed in the experimental campaign. Cross–entropy is the loss function, and the classification accuracy is evaluated over the *E* event categories. This module actually upgrades the system to a semantic interpretation of the detected events, thereby providing actionable information for higher–level monitoring and decision support.

## 5. Experimental Setup

A controlled experimental campaign in terrestrial conditions supported the evaluation of the performance of the 2-D magnetometric network. The goal of these tests was to assess the system’s ability to detect and characterize the magnetic signature of moving ferromagnetic targets, on a spatially extended sensing configuration.

### 5.1. Network Layout

**Figure 4:**
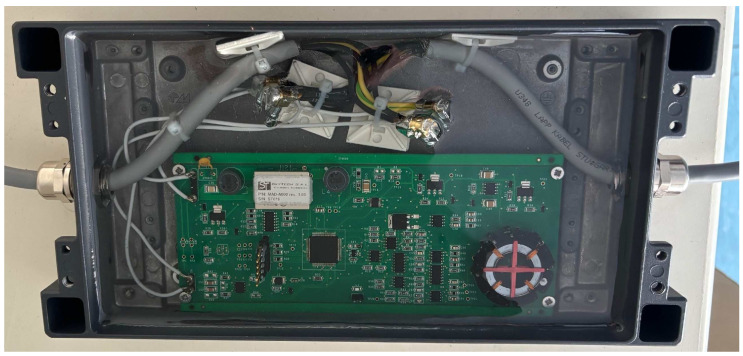
The sensing node.

The experimental setup included a 5×5 array of magnetometric sensor nodes, implementing an array of five parallel 1-D chains to form a square sensing surface. Figure 4 shows one of the sensing nodes housed in its waterproof box. The range covered by a sensing node was [−70,000; 70,000] nT at a resolution of 0.133 nT; the linearity was 0.01%.

A specifically designed supply module powered each 1-D chain; it provided a regulated voltage of 30 V with a maximum current capability of 5 A, ensuring stable operation of all sensor nodes along the chain even under varying load conditions. The power and data lines were routed together through the same cable, to simplify deployment and minimize wiring complexity throughout the test area.

The choice of a 5×5 array of magnetometric nodes was driven by a trade-off between spatial resolution and experimental complexity. From a spatial perspective, a grid of this size represents the smallest configuration that allows a genuinely two-dimensional observation of magnetic field variations, enabling the estimation of spatial gradients and the discrimination of different target trajectories across the monitored area. At the same time, increasing the number of nodes would have significantly raised the complexity of the experimental setup, including cabling, synchronization, data management.

It is worth noting that the adopted layout does not represent a limitation of the proposed approach. Owing to the modular design of the sensing chains, the network can be readily scaled to larger grids or adapted to different geometries, depending on the desired coverage and resolution requirements of a specific application.

### 5.2. Data Collection

The network covered an area of approximately $12\times 12\;m^{2}$, with uniform spacing between adjacent nodes to ensure homogeneous spatial sampling. Each node was connected to the central acquisition unit through its respective chain cable, maintaining synchronized and continuous data transmission across the entire array.

Cylindrical ferromagnetic objects provided the magnetic targets, to emulate the magnetic signature of moving metallic bodies. The tests were conducted on flat terrain, with the cylinders manually moved along predefined paths over the sensor network. The movement height was kept approximately constant (about 90 cm) to ensure consistent magnetic coupling conditions across all experiments. During all experiments, the ferromagnetic cylinder was manually moved along the predefined trajectories at approximately constant speed. The adopted motion speed was kept deliberately low and comparable to that of a very-slow-walking person, in order to ensure smooth magnetic signatures and repeatable temporal patterns across different trials.

**Figure 5:**
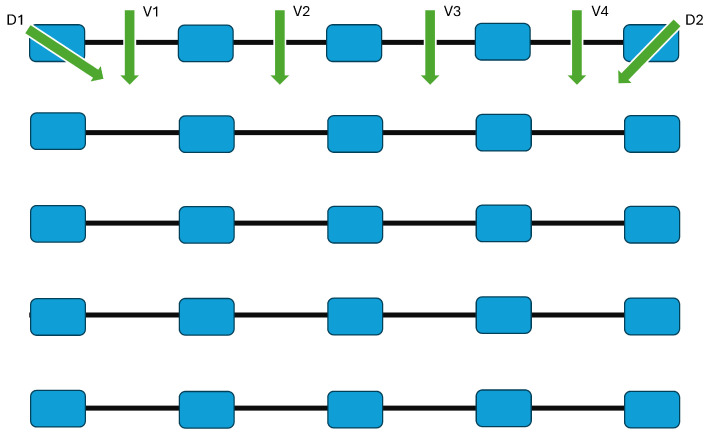
Top view of the test area showing the target trajectories. Four vertical passes (V1–V4) and two diagonal passes (D1, D2) were performed over the 5 × 5 sensor grid.

The analysis considered two main motion patterns:

- **Vertical straight-line passes:** Four trajectories were defined, each corresponding to a rectilinear movement of the target along the vertical direction of the sensing grid. These passes covered different positions, as shown in Figure 5, to evaluate the spatial response of the network to events occurring along different sectors of the sensor network.
- **Diagonal passes:** Two additional trajectories were executed along the two main diagonals of the grid—from the upper-left to the lower-right corner and from the upper-right to the lower-left corner. These tests assessed the network’s ability to track targets moving obliquely with respect to the primary sensor axes.

During all tests, each node continuously sampled the magnetic field components at a fixed sampling rate, with synchronized timestamps across the network. The recorded data streams were transmitted to the central unit, which handled real-time logging. The system acquired magnetic field data from all 25 nodes simultaneously, effectively generating a 5×5 magnetic field matrix every 30 ms. This corresponds to a temporal sampling rate of approximately 33 Hz, which was found to be adequate for capturing the magnetic transients associated with the motion of the ferromagnetic targets while maintaining synchronized acquisition across the network. The raw data acquired by the sensors were filtered as in [1]: the filtering removed the background level—which does not carry useful information—and some slow environmental variations. Eventually, one can obtain the variations in the magnetic field rather than its absolute value. As stated in Section 4, the continuous data streams acquired from the sensor network were segmented into samples using sliding temporal windows of length *T* to generate input data for the AI models. Consecutive samples were generated by shifting the window by one time step, resulting in overlapping input tensors.

Each predefined trajectory was repeated ten times during the experimental campaign in order to assess the repeatability of the acquired magnetic patterns. All repetitions were performed under comparable conditions in terms of target height, motion speed, and environmental background.

As a reference, additional measurements were also performed in the absence of any moving target, to record the environmental magnetic background and to evaluate the intrinsic noise level of the system. These “no-event” acquisitions characterized the coherent background magnetic noise and established the baseline data for subsequent anomaly detection.

**Figure 6:**
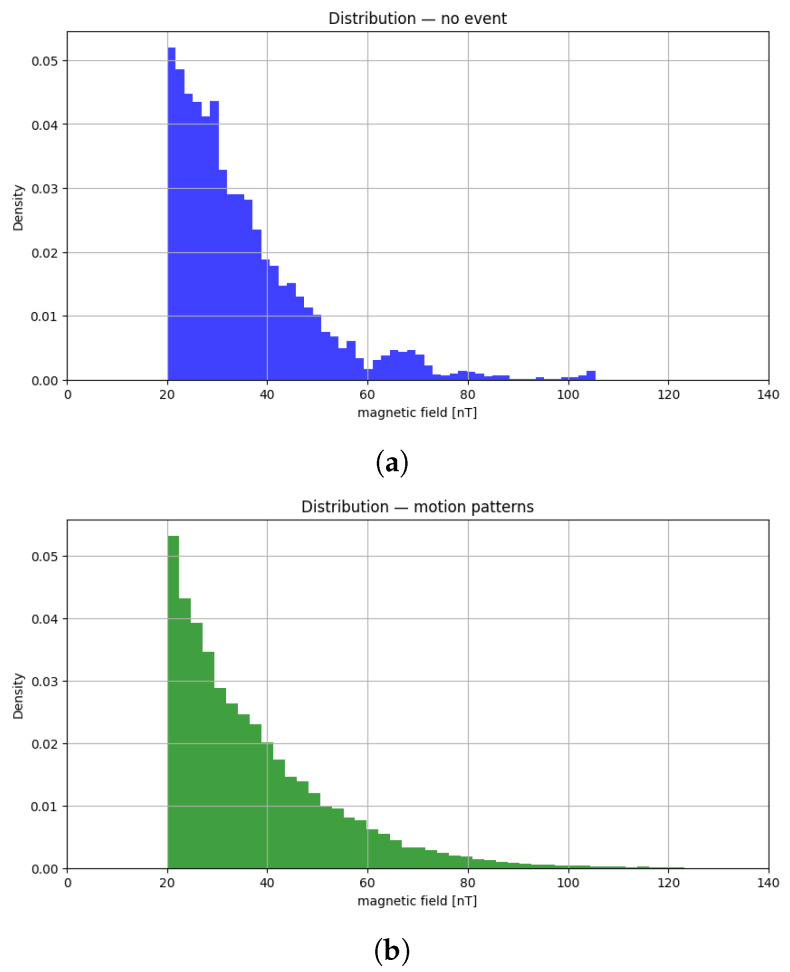
Statistical distribution of the measured magnetic field values: (**a**) no-event condition; and (**b**) samples collected during event sequences.

To provide an overview of the statistical distribution of the magnetic field values, Figure 6 gives two histograms: one corresponds to the no-event condition, whereas the other aggregates all samples collected during event sequences (i.e., all trajectories traversed by the target). In both cases, the horizontal axis marks the magnetic field intensity (in nT), and the vertical axis reports the empirical density.

For clarity of presentation, values in the range [0, 20] nT are intentionally omitted from the plots. Although they represent the most common measurements recorded by the sensor network, their very high frequency would dominate the visual scale of the graphs and obscure the differences across the remaining range, where anomalies and target-induced variations are more evident. By excluding this interval, the figures highlight the tails of the distributions and the contrasting behaviors between background noise and event-related perturbations.

The dataset obtained from these experiments provided the basis for the subsequent analysis and evaluation of the AI-based network’s detection and tracking performance, described in Section 6.

## 6. Experimental Results

### 6.1. Anomaly Detection

In the first experiment, the anomaly detection network was compared with the LAMA model [1]. The autoencoder for anomaly detection was implemented as described in Table 1. The training set contained 4588 samples, obtained from the data collected in the no-event state with T=3 (as per Section 4). The testing set held 13,353 samples, 11,668 of which corresponded to anomalies. The network was trained for 200 epochs with early stopping based on the validation loss and a patience of 15. The learning rate was set to $10^{-4}$.

**Figure 7:**
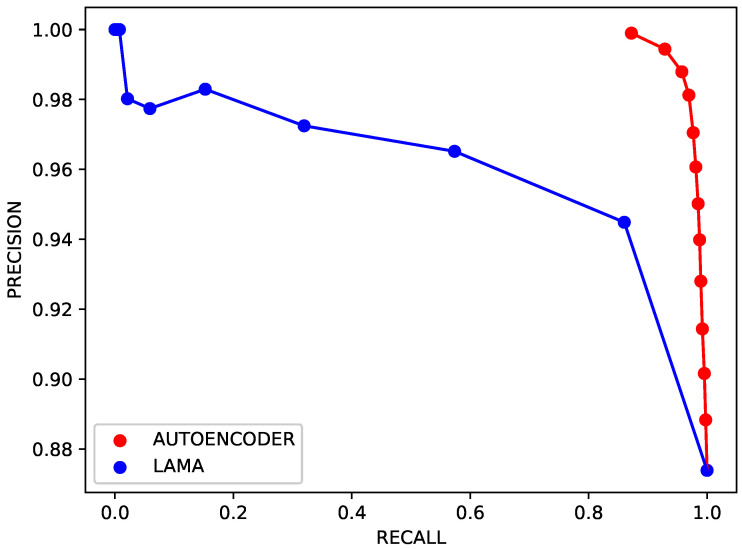
Precision/recall curves for the proposal and the baseline.

The original detection algorithm as per [1] was extended to a 2-D network, considering the relationships between all nodes within the grid. Figure 7 shows the performance of both methods on the testing data, i.e., data that were never used during the tuning of any network parameters. The precision–recall curve is given for both approaches: the red curve refers to the proposed anomaly detection model, and was obtained by varying the threshold value $t_{nor}$ in the range from the 1st to the 99th percentile. The blue curve refers to the LAMA model; the curve was obtained by varying the threshold that leads to an alert in the range from the minimum possible value to the maximum possible value according to the collected data.

The trends show a substantial difference between the proposed method and the baseline LAMA. The precision–recall comparison marked a significant discrepancy between the two approaches: the recall performance by the LAMA approach kept below 60% for most threshold values, whereas the autoencoder—across all possible configurations—always yielded both performance indicators above 87%. The proposed autoencoder-based method consistently achieves both high precision and high recall, indicating that it is able to detect magnetic anomalies while maintaining a low false-alarm rate. In contrast, the LAMA approach exhibits a markedly lower recall for most threshold values, revealing a limited capability to detect a significant fraction of target-induced events without substantially increasing the number of false positives. This behavior can be attributed to two main factors. First, the two-dimensional spatial layout provides richer information than a one-dimensional configuration, enabling the network to better discriminate localized magnetic disturbances from coherent background variations. Second, the data-driven nature of the autoencoder allows the model to learn the intrinsic structure of normal magnetic patterns directly from data, rather than relying on predefined thresholds or handcrafted features. From an operational standpoint, the improved precision–recall trade-off implies a more reliable detection capability, particularly in scenarios where missed detections are critical and false alarms must be minimized. These results confirm that the combination of a 2-D sensing architecture and AI-based processing substantially enhances anomaly detection performance with respect to the state-of-the-art baseline.

**Figure 8:**
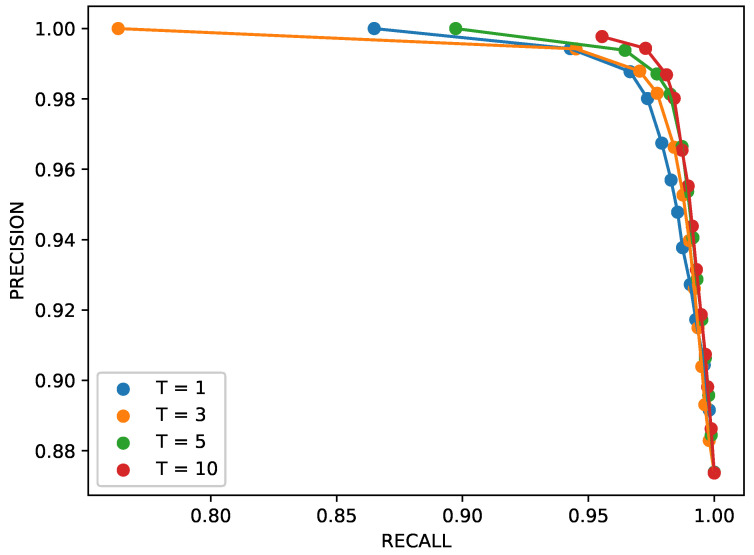
Precision/recall trends for different values of the sequence length *T*.

The impact of parameter *T* on the model’s performance is analyzed in Figure 8, giving the precision–recall curves for four settings of that hyperparameter: T∈{1,3,5,10}. The analysis showed that, except for extreme values of $t_{nor}$, the sequence length had a minor impact on the network’s behavior. Nevertheless, small performance improvements could still be observed for longer sequences. This behavior suggests that the most informative features for anomaly detection are primarily spatial rather than temporal, and that short temporal windows are sufficient to capture the magnetic signature of a moving target within the 2-D grid.

**Figure 9:**
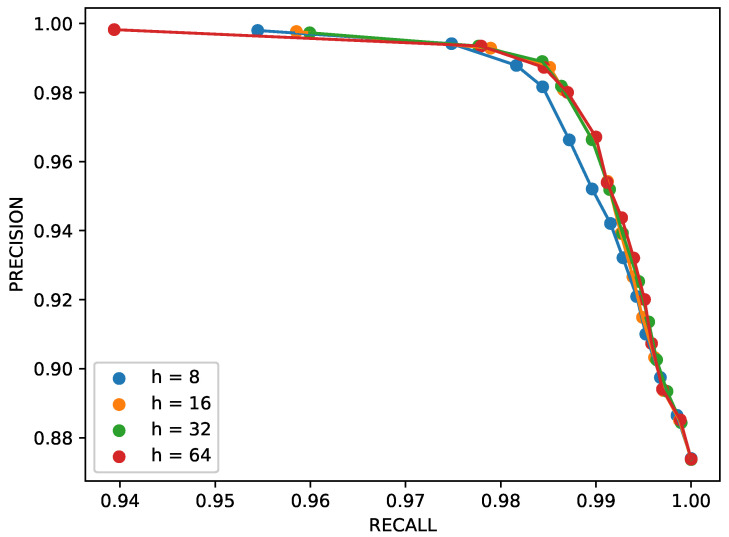
Precision/recall trends for different values of the latent space size *h*.

Figure 9 considers the impact of *h*, i.e., the dimension of the latent space. Here, four instances of the autoencoder are compared. Again, this parameter did not seem to affect the network performance significantly. The smallest considered projection, h=8, proved slightly less effective, possibly due to an excessive compression rate. The limited sensitivity to the latent-space dimension indicates that the magnetic patterns generated by the experimental targets lie on a low-dimensional manifold. This confirms that the adopted autoencoder architecture is not overparameterized and supports its suitability for embedded or real-time implementations.

**Figure 10:**
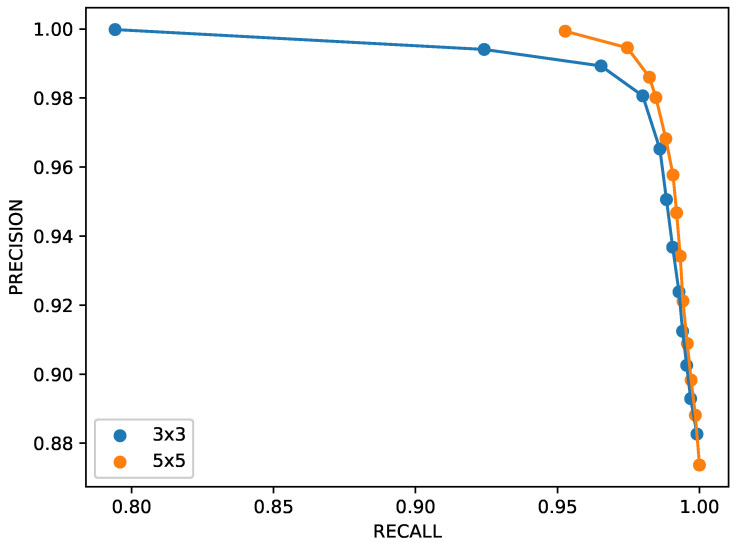
Precision/recall trends for two different configurations of the sensors network: 3×3 and 5×5.

To assess the impact of sensor density on anomaly detection performance, an additional experiment was conducted by considering a reduced 3×3 sensor grid covering the same 12×12 m^2^ area. The resulting precision–recall trends are presented in Figure 10. The figure shows that the proposed anomaly detection model maintains high performance even with a sparser spatial sampling. This result suggests that, for the considered experimental conditions, the method is robust with respect to moderate reductions in sensor density.

### 6.2. Event Classification

The classification network was tested on the task illustrated in Figure 5. A total of seven classes characterized the classification problem: NO (no-event), V1, V2, V3, V4, D1, and D2. The dataset contained 6400, 4101, and 2135 samples for training, validation, and testing, respectively; the three sets were obtained by applying a sequence length T=10 in the processing of the collected data. The seven categories were roughly equally distributed in the dataset.

**Figure 11:**
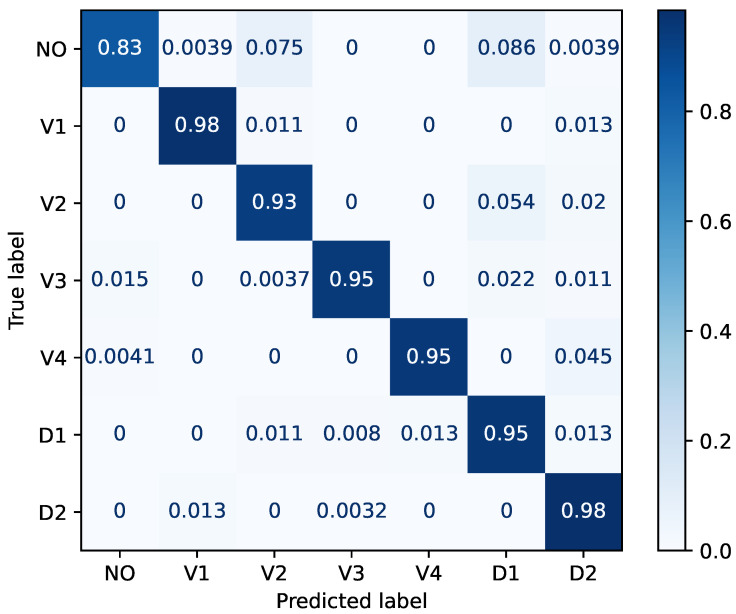
Confusion matrix for the task proposed in Figure 5.

The network described in Section 4.2 was trained for 200 epochs with early stopping based on the validation loss, by applying a patience setting of 15. The learning rate was $10^{-4}$. Figure 11 shows the confusion matrix obtained on the testing dataset for the seven-class event classification task. The matrix provides a detailed view of the classifier behavior, highlighting both correct predictions and misclassification patterns across different trajectories. Most classes are recognized with high accuracy, confirming that the spatial information captured by the 2-D magnetometric network is sufficient to discriminate between different target paths. Misclassifications mainly occur between spatially adjacent trajectories, which is consistent with the smooth spatial variation in the magnetic field and the finite spatial resolution of the sensor grid. This behavior reflects a physical limitation of the sensing configuration rather than a deficiency of the learning model.

It is important to remark that the event classification module is not intended to operate as a stand-alone detector. Instead, it represents a second semantic stage in a hierarchical processing pipeline, which is activated only after the anomaly detection module has identified the presence of a significant magnetic disturbance. In this two-stage configuration, the anomaly detector acts as a robust gatekeeper that filters out background noise and no-event conditions, while the classifier focuses exclusively on the interpretation of confirmed events.

From an operational perspective, this separation offers several advantages. First, it significantly reduces the impact of occasional misclassifications of the no-event class observed in Figure 10, as the classifier would not be invoked in the absence of an anomaly. Second, it allows the two models to be optimized independently: the anomaly detector can prioritize sensitivity and robustness, whereas the classifier can focus on extracting spatial and directional information from short event sequences.

The results reported in this section confirm the effectiveness of this hierarchical approach. Even under a limited experimental setup, the classifier achieves high accuracy in distinguishing different target trajectories once an event has been detected. This demonstrates that the proposed 2-D architecture provides sufficient spatial information to support not only detection but also semantic interpretation of magnetic events, paving the way toward more advanced tracking and decision-support functionalities.

## 7. Discussion

The presented results demonstrate that the LAMA system can be effectively extended from its original one-dimensional (1-D) configuration to a fully operational two-dimensional (2-D) sensing architecture. This transition significantly enhances the quality and richness of the information that can be extracted from magnetic field measurements. In particular, the 2-D layout is advantageous when the objective is not only detection but also the extraction of spatially structured information (e.g., motion direction and coarse trajectory estimation), and when the monitored area is wide or geometrically complex. However, a 2-D network inevitably increases deployment and operational complexity, including cabling, synchronization, installation effort, and data handling. In contrast, a 1-D architecture may remain preferable for barrier-like surveillance tasks along a boundary or a corridor (e.g., narrow passages, entrances, or perimeter lines), where the main goal is reliable detection with minimal installation complexity and reduced system cost.

A second major outcome concerns the integration of artificial intelligence models into the processing pipeline. By combining a spatially extended sensing grid with data-driven approaches, the system moves beyond classical threshold-based logic traditionally adopted in magnetic anomaly detection. Both anomaly detection and event classification are achieved by learning spatio-temporal patterns directly from data, improving robustness against non-stationary background noise and reducing dependence on manually tuned parameters.

The event classification task considered in this study relies on a discretization of target motion into a limited set of predefined trajectories. This choice represents a simplified experimental setting, adopted to validate the feasibility of trajectory discrimination using the proposed 2-D magnetometric architecture and AI-based processing pipeline. It is not intended to imply that real targets are constrained to a small number of ideal paths. The selected vertical and diagonal trajectories were chosen to excite different spatial patterns across the sensing grid while keeping the experimental protocol manageable in a preliminary evaluation. Although horizontal linear motion was not explicitly included in the experimental campaign, the two-dimensional sensor layout is symmetric with respect to rotations of the spatial reference frame. As a result, horizontal and vertical motions are expected to generate analogous magnetic signatures, up to a rotation of the observed spatial patterns. Nevertheless, extending the experimental validation to include horizontal and more complex trajectories is an important direction for future work. Such extensions will be necessary to assess the robustness and generalization capabilities of the classification model, and to move beyond discrete path categorization toward continuous trajectory estimation and tracking.

When compared with alternative sensing and detection technologies, the proposed 2-D magnetometric system exhibits a number of distinctive advantages as well as inherent limitations. Unlike acoustic or optical surveillance systems, magnetic sensing is largely insensitive to water turbidity, lighting conditions, and acoustic clutter, making it particularly suitable for environments where visibility is poor or background noise is highly variable. Moreover, the adoption of a two-dimensional spatial layout enables the extraction of directional and trajectory-related information, which is typically unavailable in conventional one-dimensional magnetic barriers or point-sensor configurations.

The integration of data-driven AI models further differentiates the proposed approach from traditional magnetic anomaly detection methods based on thresholding or handcrafted signal processing rules. By learning spatio-temporal patterns directly from data, the system adapts more naturally to complex and non-stationary background conditions, reducing the need for manual parameter tuning and improving robustness in realistic scenarios.

On the other hand, the proposed system also presents some limitations. Magnetic sensing is inherently sensitive only to ferromagnetic objects, which restricts its applicability to targets that produce a sufficiently strong magnetic signature. In addition, the effective monitoring area is directly related to the density and spatial distribution of the sensor nodes, implying a trade-off between coverage, spatial resolution, and deployment complexity. These aspects should be carefully considered when comparing the proposed solution with wide-area acoustic or radar-based systems.

A further design aspect that deserves discussion concerns the choice of measuring only two components of the magnetic field at each sensing node. This decision reflects a deliberate trade-off between information content and system complexity. Restricting the sensor to dual-axis measurements simplifies the electronic design, reduces calibration effort, and lowers power consumption, which are critical factors in large-scale distributed networks. From an information-theoretic perspective, omitting the third magnetic field component may reduce directional sensitivity in complex three-dimensional scenarios. However, previous studies on magnetometric barriers for intrusion detection have shown that reliable detection performance can be achieved using two-axis configurations when sensors are spatially distributed. In such settings, spatial redundancy across the network partially compensates for the reduced dimensionality at the single-node level. In the present work, the same design choice was retained to ensure consistency with existing systems and to focus on validating the proposed 2-D architecture and AI-based processing pipeline under controlled conditions. Nonetheless, the proposed framework does not inherently limit the sensing nodes to dual-axis operation. Extending the system to full three-axis measurements represents a natural direction for future research, particularly in scenarios involving complex target motion or requiring enhanced localization accuracy.

Although the experimental validation focused on intrusion detection scenarios inspired by underwater surveillance, the proposed architecture is intrinsically domain-agnostic and lends itself to a broad range of alternative applications. For instance, land-based security and perimeter protection represent a natural extension: a 2-D magnetometric grid could be deployed to monitor restricted areas, border zones, or critical infrastructures, enabling the detection and tracking of unauthorized vehicles or individuals carrying metallic equipment, even in visually occluded or low-visibility conditions.

Despite the relatively modest size of the dataset used in this study, the AI models achieved promising performance in both anomaly detection and event classification. This suggests that the problem exhibits a favorable spatio-temporal structure that convolutional architectures can exploit efficiently. Larger and more diverse datasets—covering different deployment geometries, environmental conditions, and target typologies—are therefore expected to further improve generalization and robustness.

Overall, the results presented here lay the groundwork for a new phase in the evolution of magnetometric sensor networks. Future work will include both extended terrestrial experiments in heterogeneous environments and in-water validation campaigns, with the long-term goal of developing a versatile, intelligent, and scalable 2-D magnetic sensing platform applicable across multiple operational domains.

## 8. Conclusions

The work presented in this paper set out to investigate two main objectives: the feasibility of extending the LAMA system from a 1-D to a 2-D sensing architecture, and the integration of data-driven models to enhance event detection and classification capabilities. Both objectives have been successfully addressed.

The proposed 2-D architecture was designed, implemented, and experimentally validated, demonstrating that a planar network of magnetometric nodes can operate coherently and provide spatial information that is unattainable with a 1-D configuration. The experiments carried out on land confirm the system’s ability to detect and characterize magnetic disturbances generated by a moving ferromagnetic target.

Furthermore, the adoption of deep learning models has proven effective in enabling both unsupervised anomaly detection and supervised event classification. These results show that the combination of structured magnetic data from a 2-D grid and AI-based processing constitutes a viable path toward adaptive, threshold-independent monitoring solutions.

Overall, the outcomes of this study provide a solid foundation for future developments, which will include extended experimental campaigns and the transition toward in-water testing under realistic operational conditions.

## Figures and Tables

**Table 1:** Architecture of the autoencoder adopted for anomaly detection.

Block	Layer	Parameters
Encoder	Conv3D	$f_{s}$ = 8, $k_{s}$ = (3,3,3), activation = ‘relu’
	BatchNormalization	
	MaxPooling3D	(2,2,1)
	Conv3D	$f_{s}$ = 4, $k_{s}$ = (3,3,3), activation = ‘relu’
	BatchNormalization	
	Flatten	
Latent Representation	Dense	$h=L_{DIM}$, activation = ‘relu’
Decoder	Dense	$n_{h}=3\times 3\times T\times 4$, activation = ‘relu’
	Reshape	(3,3,T,4)
	Conv3D	$f_{s}$ = 4, $k_{s}$ = (3,3,3), activation = ‘relu’
	UpSampling3D	(2,2,1)
	Conv3D	$f_{s}$ = 8, $k_{s}$ = (3,3,3), activation = ‘relu’
	Conv3D	$f_{s}$ = 1, $k_{s}$ = (3,3,3), activation = ‘sigmoid’

## Data Availability

The raw data supporting the conclusions of this article will be made available by the authors on request.
